# 1278. Real World Treatment Experience of Single Tablet Dolutegravir/Lamivudine in Those Naïve to Treatment with Baseline Viral Loads ≥ 100,000 copies/mL in the US

**DOI:** 10.1093/ofid/ofac492.1109

**Published:** 2022-12-15

**Authors:** Paul Benson, Cindy Donovan, Gavin Harper, Deanna Merrill, Katie L Mycock, Alan Oglesby, Jimena Patarroyo, Aimee Metzner

**Affiliations:** Be Well Medical Center, Berkley, Michigan; ViiV Healthcare, Research Triangle Park, North Carolina; Adelphi Real World, Bollington, England, United Kingdom; ViiV Healthcare, Research Triangle Park, North Carolina; Adelphi Real World, Bollington, England, United Kingdom; ViiV Healthcare, Research Triangle Park, North Carolina; ViiV Healthcare, Research Triangle Park, North Carolina; ViiV Healthcare, Research Triangle Park, North Carolina

## Abstract

**Background:**

Treatment for people living with HIV-1 (PLWH) continues to advance with a two-drug regimen (2DR) approach. Dolutegravir/lamivudine (DTG/3TC) is indicated as a 2DR for both treatment-naïve and virally suppressed PLWH. Despite high and sustained virologic efficacy for DTG-based 2DRs observed in clinical trials, there is limited evidence in US real world clinical settings. This study characterizes prescribing behaviors and treatment outcomes for DTG-based 2DR in the real world.

**Methods:**

TANDEM was a retrospective medical chart review conducted across 24 US sites. Eligible PLWH were adults initiated on single tablet DTG/3TC or DTG/rilpivirine prior to Sept/30/2020 with a minimum clinical follow-up of six months. Treatment-naïve PLWH had no prior history of HIV therapy. Clinical characteristics, treatment history and outcomes were abstracted. Analyses were descriptive. Reported here are viral outcomes for the DTG/3TC cohort of treatment-naïve PLWH with baseline viral loads (VLs) ≥ 100,000 (c/mL).

**Results:**

From an overall sample of 469 PLWH on DTG-based 2DR, 318 received DTG/3TC. Of the DTG/3TC cohort, 126 were treatment-naïve. Of the treatment-naïve, 58 PLWH had known VLs available at DTG/3TC initiation. For those with baseline VLs ≥ 100,000 c/mL, 9 had values 100,000-250,000 c/mL while 7 were > 250,000 c/mL. Characteristics of this sub-cohort are described in Table 1. Overall, the most common reason for DTG/3TC initiation in this sub-cohort was patient preference (n=5), followed by avoidance of long-term toxicities and convenience (both n=3). For those with VLs between 100-250k, median CD4 count was 312 while 8/9 became virally suppressed (HIV-1 RNA < 50c/mL) and 1 PLWH had missing data. For those with VLs > 250k, median CD4 count was 114 while 6/7 became virally suppressed and 1 PLWH had missing data. One of these 6 PLWH experienced virologic rebound yet remained on DTG/3TC.

**Table 1:**
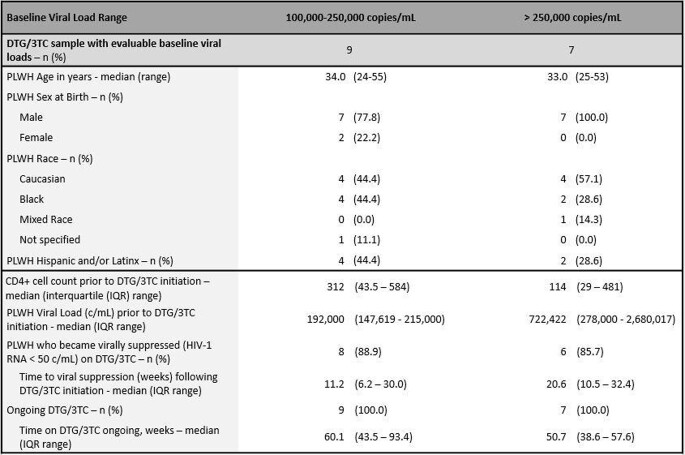
Clinical Characteristics

**Conclusion:**

These real world results reflect data from clinical trials, demonstrating DTG/3TC is effective and well tolerated in the real world. Nearly all DTG/3TC users, regardless of baseline VL, experienced sustained virologic suppression with few treatment discontinuations. Caution should be used when extrapolating these results due to limited population size of the sub-cohorts.

**Disclosures:**

**Paul Benson, DO, AAHIVS**, ViiV Healthcare: Advisor/Consultant|ViiV Healthcare: Speakers Bureau and Advisory Boards **Cindy Donovan, PharmD**, Johnson & Johnson: Stocks/Bonds|ViiV Healthcare: Employee/Salary|ViiV Healthcare: Stocks/Bonds **Gavin Harper, BA**, ViiV Healthcare: Adelphi Real World were paid consultants (CRO) to conduct the observational research study on behalf of ViiV Healthcare.|ViiV Healthcare: Adelphi Real World were paid consultants (CRO) to conduct the observational research study on behalf of ViiV Healthcare **Deanna Merrill, PharmD, MBA, AAHIVP**, ViiV Healthcare: Salaried employee|ViiV Healthcare: Stocks/Bonds **Katie L. Mycock, MChem**, ViiV Healthcare: Adelphi Real World were paid consultants (CRO) to conduct the observational research study on behalf of ViiV Healthcare|ViiV Healthcare: Adelphi Real World were paid consultants (CRO) to conduct the observational research study on behalf of ViiV Healthcare **Alan Oglesby, MPH**, GlaxoSmithKline (GSK): Employment|GlaxoSmithKline (GSK): Stocks/Bonds **Jimena Patarroyo, PharmD, AAHIVP**, ViiV Healthcare: Salaried employee|ViiV Healthcare: Stocks/Bonds|ViiV Healthcare: Stocks/Bonds **Aimee Metzner, PharmD, AAHIVP**, ViiV Healthcare: Salaried employee|ViiV Healthcare: Stocks/Bonds.

